# Characterizing the Validity and Real-World Utility of Health Technology Assessments in Healthcare: Future Directions: Comment on "Problems and Promises of Health Technologies: The Role of Early Health Economic Modelling"

**DOI:** 10.15171/ijhpm.2019.132

**Published:** 2019-12-11

**Authors:** Nadine K. Zawadzki, Joel W. Hay

**Affiliations:** ^1^Schaeffer Center for Health Policy and Economics, Department of Pharmaceutical and Health Economics, School of Pharmacy, University of Southern California, Los Angeles, CA, USA.; ^2^USC Clinical Economics Research and Education Program (CEREP), Los Angeles, CA, USA.

**Keywords:** Health Technology Assessment, Economic Modeling, Cost-Effectiveness, Transparency

## Abstract

With their article, Grutters et al raise an important question: What do successful health technology assessments (HTAs) look like, and what is their real-world utility in decision-making? While many HTAs are published in peer-reviewed journals, many are considered proprietary and their attributes remain confidential, limiting researchers’ ability to answer these questions. Models for economic evaluations like cost-effectiveness analyses (CEAs) synthesize a wide range of evidence, are often statistically and mathematically sophisticated, and require untestable assumptions. As such, there is nearly universal agreement among researchers that enhancing transparency is an important issue in health economic modeling. However, the definition of transparency and guidelines for its implementation vary. Model registration combined with a linked database of model-based economic evaluations has been proposed as a solution, whereby registered models and their accompanying technical and nontechnical documentation are sourced into a single publicly-available repository, ideally in a standardized format to ensure consistent and complete representation of features, code, data sources, results, validation exercises, and policy recommendations. When such a repository is ultimately created, modelers will not have to reinvent the wheel for every new drug launched or new treatment pathway. These more open and transparent approaches will have substantial implications for model accuracy, reliability, and validity, improving trust and acceptance by healthcare decision-makers.

In seeking to understand the returns to early health economic modeling, Grutters et al^1^ found that assessments performed in early development led to further development and research, and that none resulted in a firm ‘no-go’ recommendation. These results suggest that the benefits of early modeling are potentially very high. With their article, Grutters et al raise an important question: What do successful health technology assessments (HTAs) look like, and what is their real-world utility in decision-making? As stated by Mandelblatt et al, “Models are only as good as their ability to represent reality at the level needed to draw useful conclusions; this, in turn depends upon their structure and on the assumptions that go in to the models.”^2^ The process of determining how ‘good’ a model is involves model validation (predictive and structural) and peer review.^3^ While there are tools for measuring the general quality of published economic models (eg, the Quality of Health Economic Studies instrument),^4^ there are no such standard tools for point-by-point evaluation of validity and applicability in peer review. How, then, can we characterize the systematic factors of HTA success and failure?

One could begin by comparing models and their predictions against available data on market and health outcomes. However, this requires access to detailed information on models. While many HTAs such as cost-effectiveness analyses (CEAs) are published in peer-reviewed journals, many are considered proprietary and their attributes remain confidential. Due to confidentiality concerns, Grutters et al were limited in what they could report; only two assessments reviewed were published in scientific journals. As a result, they were unable to identify specific characteristics contributing to decisions favoring further model development or assess the key points of success and failure.

The trade-offs between confidentiality and model transparency in CEA have been discussed in the literature for over a decade. Because CEAs synthesize a wide range of evidence, are often statistically and mathematically sophisticated, and require untestable assumptions, many key decision-makers may view models as proprietary black boxes designed to give biased results.^5^ In the United States, for example, model results are often discounted by skeptical clinicians and policy-makers.^6^ While there is nearly universal agreement among researchers that enhancing transparency is an important issue in health economic modeling,^7-9^ the definition of transparency and guidelines for its implementation vary, especially with respect to sharing model code.^10^ The Second Panel on Cost-effectiveness in Health and Medicine recommends that modelers should provide enough detail about model structure and parameterization to allow reproducibility, from making entire models available to providing versions that allow users to vary selected inputs, but makes no formal recommendation for sharing model code.^7^ The International Society for Pharmacoeconomics and Outcomes Research (ISPOR) and the Society of Medical Decision Making recommends that transparent models should provide enough information to enable readers, specialists and non-specialists alike, to understand a model’s accuracy, limitations, and potential applications.^8^ This requires non-technical documentation detailing model structure and potential applications, though not at the level of detail necessary for replication, in addition to technical documentation more substantially detailing model structure, equations, data input and, at the modeler’s discretion, access to model code to permit replication.

While some argue that open-source publication of models would compromise intellectual property rights and disincentivize their development,^11^ economic models themselves often have minimal proprietary value, and in many situations, publication in a prestigious peer-reviewed journal is worth more than the ability to sell the model privately.^6,12^ Several top scientific journals such as *Nature* , *Cell* , and *Science* now require data and/or code sharing with readers upon publication.^13^ Since most applied modeling techniques and software packages are standard among researchers in the field, model scripts and code contain little innovative intellectual property.^12^ Healthcare organizations are more likely to be concerned with the release of proprietary data from clinical trials. However, when clinical trials are biased or not adequately generalizable, those CEAs that use their results will suffer from these same limitations.

Take, for example, a CEA comparing various strategies to reduce cholesterol involving diet, niacin, and lovastatin, published in Appendix C of *Cost Effectiveness in Health and Medicine* by Gold et al,^14^ a landmark instructional book for economic modeling in healthcare. At the time of the book’s publication in 1996, clinical trials on niacin showed favorable changes in lipid profiles.^15-18^ Although it had not been established in clinical research that niacin’s effect on lipid levels resulted in lower rates of cardiovascular events, the authors assumed that such a relationship existed between lipid levels and cardiovascular risk for niacin users. Using the biomarker results, the authors of the CEA concluded that care with niacin was cost-effective for prevention of cardiovascular disease, and in 1997, the US Food and Drug Administration approved a prescription extended-release version of niacin. However, nearly two decades later, the results of two large prospective trials showed that niacin did not improve prevention or reduce mortality.^19,20^ Although the original clinical trials were presumed to show clinical effectiveness, most were inadequately powered and relied on surrogate measures that had not been formally validated. Very few reported information on cardiovascular outcomes, and even fewer were designed to detect changes in these clinical outcomes.^21^ Ultimately, the CEA published in Appendix C relied on data that were poor quality and its recommendations were invalidated when better outcome data became available. Since CEAs require synthesis of estimates from various sources, it is important that modelers communicate their assumptions and their reasons for selecting certain data sources over others. Justification for model inputs and parameters is integral to model transparency, especially when data is confidential or otherwise not publicly available. If the authors were required to publish technical documentation detailing the assumed relationship between lipid levels and cardiovascular risk, perhaps a reviewer or reader would have questioned their decision to use an unvalidated surrogate endpoint.

It is possible to build accurate and useful economic models without relying solely on clinical data. Indirect and unintended outcome measures from more pragmatic settings and registries that take into account patient heterogeneity and real-life experiences should also be considered to answer a HTA question.^22^ Real-world evidence (RWE) is used by several HTA bodies to confirm or supplement findings from clinical trials,^23^ and in certain cases could be used to demonstrate treatment effects when clinical trials are not feasible or unethical, or when there is significant unmet need. Advanced statistical and data science techniques such as Deep IV, a deep neural net machine learning algorithm for counterfactual prediction,^24^ and E-value analysis of unmeasured confounding^25^ help address bias in observational data and permit causal inferences using RWE. RWE has utility in each stage of product lifecycles, from development to market access and post-launch.^22^ Of the assessments reviewed by Grutters et al, 23 were constructed without clinical data in early stages of development, all of which led to further development, research, or both.^1^ As with clinical trial data, transparency in analyses of RWE would further strengthen HTA insights gained.

Despite agreement on the importance of transparency in health economic modeling, there is no definitive method for assessing transparency. However, all major guidelines emphasize reproducibility in their recommendations. Some argue that replication studies can effectively demonstrate how transparently a model is reported, identify potential calculation errors and inform future reporting practices.^26^ The most recent paper to discuss the concept of a successful replication reported on the 8th Mt. Hood Diabetes Challenge Network: Economics, Simulation Modelling and Diabetes Competition,^27^ at which researchers compare their health economic diabetes models in terms of their structure and performance through coordinated tests in which they are asked to reproduce a real-world result.^28^ Through the competition’s process, the researchers present their models in greater detail than typically provided in publications, and may compare data sources, underlying code, and calibration techniques. In the competition, the replication challenge was used to indicate reporting transparency, and their proposed definition of replication success was described in terms of model transparency.^27^ Recently, the Mt. Hood Diabetes Challenge Network has developed formal guidelines and a checklist, published in *Value in Health,*^27^ to further improve transparency in reporting of input data and other information underlying model-based analyses for diabetes.

In 2018, Shao et al participated the 9th Mt. Hood Challenge to demonstrate that their BRAVO microsimulation model of diabetes costs and outcomes,^29^ based on the publicly available ACCORD patient-level clinical trial data, performs much better on many, if not all, dimensions than other advanced diabetes models, all of which are based on the much older UKPDS data.^30^ In development of BRAVO, Shao et al devised a novel approach to estimate regional multipliers for diabetes models that are constructed using data from a single region, thereby improving prediction accuracy by reducing systematic bias and increasing explanation power.^31^ Typical methods for external validation comparing model predictions to outcomes from single-region clinical trials may only weakly assess Shao et al’s new approach. However, the Mt. Hood Challenge provided Shao et al a unique opportunity to validate their approach against that of their competitors, permitting them to thoroughly identify inconsistencies and determine their source, whether it be the data and/or model calibration.

Of course, conferences and competitions are not feasible for every systematic evaluation of HTAs. Model registration combined with a linked database of model-based economic evaluations has been proposed as a solution, whereby registered models and their accompanying technical and nontechnical documentation are sourced into a single publicly-available repository, ideally in a standardized format to ensure consistent and complete representation of features, code, data sources, results, validation exercises, and policy recommendations.^32^ Such a registry would aid researchers and decision-makers who are interested in comparing HTAs, their characteristics, and their relative performance and accuracy to examine and factor all available information into their evaluations. A registry would additionally facilitate collaboration between researchers while avoiding duplicating efforts to answer certain policy questions or validate existing models. ISPOR has already convened a Special Interest Group that is currently undertaking development of such an open-source platform. In the last year, it has developed tools for the ISPOR website, Short Courses, manuscripts in draft for scientific journals, webinars, and presentations in order to promote open-source modeling. The Mount Hood Diabetes Challenge Network has also created an initial registry for diabetes models in response to recent calls for a registry of economic models, located on their website.^33^ While their registry does not require model code to be made available and is currently limited to non-technical documentation, with outside links to technical descriptions and published reports, the Network is currently establishing a structured form to house technical and non-technical documentation of contributing models.^32^ When a repository is ultimately created, modelers will not have to reinvent the wheel for every new drug launched or new treatment pathway.

Grutters et al were disinclined to report the point estimate results of assessments in terms of expected costs, effects, or cost-effectiveness without proper explanation of the context of analysis and quality of underlying evidence. An open-source repository of models would make this information publicly available through accompanying documentation, permitting Grutters et al to critically examine these results in their study. Assuming that technical documentation would also include probabilistic and scenario analyses beyond that typically reported in published manuscripts, Grutters et al could have additionally commented on existing uncertainty surrounding care. Although Grutters et al have presented many important insights, they caution that their recommendations cannot be interpreted independently as they are strongly related to both the value proposition and positioning of products. If a model repository were available, Grutters et al could have compared their included HTAs to those performed for competitor products at various stages of development, supplementing their sample so that it no longer represents only those innovations for which an early health economic modeling study was commissioned, thereby further informing value, positioning, and commercial viability. These more open and transparent approaches will have substantial implications for model accuracy, reliability, and validity, improving trust and acceptance by healthcare decision-makers. As Grutters et al aimed to demonstrate, evaluating the real-world performance of such models would promote more efficient and effective healthcare HTA.

## Ethical issues

Not applicable.

## Competing interests

Authors declare that they have no competing interests.

## Authors’ contributions

Both JWH and NKZ contributed to the conception, drafting, and review of this commentary.

## Authors’ affiliations

^1^Schaeffer Center for Health Policy and Economics, Department of Pharmaceutical and Health Economics, School of Pharmacy, University of Southern California, Los Angeles, CA, USA. ^2^USC Clinical Economics Research and Education Program (CEREP), Los Angeles, CA, USA.
